# 0468. Cerebral effects of lateral trendelenburg vs semirecumbent position in an experimental model of ventilator-associated pneumonia

**DOI:** 10.1186/2197-425X-2-S1-O14

**Published:** 2014-09-26

**Authors:** J López-Aguilar, G Li Bassi, ME Quílez, JD Martí, M Rigol, O Tavares-Ranzani, E Aguilera, I Ferrer, L Blanch, A Torres

**Affiliations:** Fundació Parc Taulí, Sabadell, Spain; CIBERES, Madrid, Spain; Instituto del Torax, Hospital Clinic, IDIBAPS, UB, Barcelona, Spain; Idibell, Hospital Universitario de Bellvitge, L'Hospitalet de Llobregat, Spain; CIBERNED, L'Hospitalet de Llobregat, Spain; Corporació Sanitária i Universitária Parc Taulì-UAB, Sabadell, Spain

## Introduction

Lateral-Trendelenburg position has been postulated as a promising intervention to prevent ventilator-associated pneumonia (VAP) because it improves mucus clearance and avoids pulmonary aspiration in intubated patients [1]. Mechanical ventilation (MV) per se can modify brain status [2, 3] but the effects of position during MV are unknown.

## Objectives

To investigate the preventive effects of lateral Trendelenburg (TL) vs semirecumbent (SR) position in the development of VAP and its effects in the brain in an experimental model in pigs.

## Methods

We have studied 17 Large White-Landrace pigs (30±2 kg) anesthetized, intubated and MV during 72 h in volume control. Animals were randomized in 3 groups:

1. SR: SR position, PEEP 0cmH2O, inspiratory-expiratory ratio (I:E) 0.33,
2. SR-inv: SR position, PEEP 5 cmH2O, I:E 0.7, and
3. TL: TL position, PEEP 0cmH2O, and I:E 0.33.

All the animals were oropharyngeal instilled (10ml) with *P aeruginosa* (10^7^-10^8^ cfu/ml). Mean arterial pressure (MAP) was monitorized and final bacterial pulmonary colonization, and cerebral status, consisting in a macroscopical evaluation of haemorrhage and of apoptosis indicatiors (caspase and TUNEL) in dentate gyrus in the hippocampal formation, were evaluated.

## Results

Bacterial Colonization (0.22 vs 2.27 and 2.31 log cfu/gr, p< 0.05) and VAP (0 vs 67 and 86 %, p< 0.05) were drastically reduced in TL position as compared to SR and SR-inv respectively. MAP was lower in SR and SR-inv compared to TL position (80 and 73 vs 90 mmHg, p< 0.05). At the brain level, pigs in TL position presented high score of petequial hemorrhage (2.6 vs 1 and 1.5, p< 0.05), and higher levels of immunopositive cells to caspase (6.3 vs 2.5 and 1.7, p< 0.05) and TUNEL (5.17 vs 1 and 2.72, p< 0.05) in the dentate gyrus in the hippocampus, both indicators of apoptosis, in comparison with groups SR and SR-inv respectively.

## Conclusions

In this pig model of MV, TL position prevents pulmonary colonization and VAP development, but enhances cerebral hemorrhage, and increased apoptosis in the hippocampus. These alterations in the brain could be related with the increase in MAP observed in TL position. More studies to evaluate risks and benefits of TL position are needed.
